# Alternative Approach to Design and Optimization of High-Q Ring Resonators for Membrane-Free Acoustic Sensors

**DOI:** 10.3390/mi14101876

**Published:** 2023-09-29

**Authors:** Yongqiu Zheng, Jiamin Chen, Yuan Han, Jiandong Bai, Yifan Luo, Yonghua Wang, Chenyang Xue

**Affiliations:** State Key Laboratory of Dynamic Measurement Technology, North University of China, Taiyuan 030051, China; zhengyongqiu@nuc.edu.cn (Y.Z.); hanyuan96688@163.com (Y.H.); jdbai@nuc.edu.cn (J.B.); luoyifan119@163.com (Y.L.); wangyonghua@nuc.edu.cn (Y.W.); xuechenyang@nuc.edu.cn (C.X.)

**Keywords:** acoustic sensor, optical waveguide ring resonator, membrane-free, wide-frequency response, high sensitivity

## Abstract

Membrane-free acoustic sensors based on new principle and structure are becoming a research hotspot, because of many advantages, e.g., their wide bandwidth and high sensitivity. It is proposed that a membrane-free acoustic sensor employs a semi-buried optical waveguide ring resonator (SOWRR) as a sensing element. Using air as the upper cladding medium, the excited evanescent field in the air cladding medium would be modulated by acoustic wave. On this basis, the acoustic sensing model is established. Taking high Q factor and resonance depth as design criteria, the optimal design parameters are given. The optimal values of the air/SiO_2_: Ge/SiO_2_ waveguide resonator length and coupling spacing are obtained as 50 mm and 5.6 μm, respectively. The Q factor of the waveguide resonator of this size is as high as 8.33 × 10^6^. The theoretical simulation indicates that the frequency response ranges from 1 Hz to 1.58 MHz and that the minimum detectable sound pressure is 7.48 µPa using a laser with linewidth of 1 kHz. Because of its advantages of wide bandwidth and high sensitivity, the membrane-free sensor is expected to become one of the most promising candidates for the next-generation acoustic sensor.

## 1. Introduction

Compared with traditional electroacoustic sensors, optical acoustic sensors have the advantages of anti-electromagnetic environment interference [1,2]. Furthermore, optical membrane-free acoustic sensors, due to the lack of moving parts, have excellent performance in terms of their wide bandwidth and high sensitivity compared to those of traditional acoustic sensors with a membrane [3,4,5,6]. The optical membrane-free acoustic sensor has great application prospects and has seen great market demand in both scientific research and industrial fields [7,8,9,10]. For this reason, it has received great attention from researchers worldwide. It is expected to become one of the most promising candidates for the next-generation acoustic sensor.

In recent years, as an emerging technology, membrane-free optical acoustic sensorss have become rid of the defects caused by movable parts [3,11]. In 2016, XARION Laser Acoustics described an all-optical membrane-free acoustic sensor [3,12]. The sensing structure is a rigid Fabry–Perot etalon (FPE) composed of two partially transmitted flat mirrors. The frequency response range of the acoustic sensor in air is 10~1 MHz, the dynamic range is 100 dB, and the sensitivity is 10 mV/Pa. Additionally, its fabrication process is complicated, which has brought challenges to batch and consistency in production. The Mach–Zehnder interferometer membrane-free fiber-optic acoustic sensor (FOAS) achieves a frequency response of 500 Hz to 20 kHz and a sensitivity of 77 mV/Pa [4]. However, its sound-sensitive structure is the air cavity formed via the alignment and coupling of a pair of collimators, which has the disadvantages of a poor mechanical stability, large size and inconvenient integration. Other research groups have achieved ultrasound detection using polymer or microbubble microring resonators for photoacoustic imaging or underwater acoustic wave detection [13,14,15,16,17]. Although the volume of microring resonators can be very small, the quality factors are in the order of 10^5^. Moreover, the resonators have greater optical loss and their reliability needs to be improved.

For the batch, consistency and single-chip integration of sensors [18,19], optical waveguide ring resonator (OWRR) technology has developed rapidly over the past few years [20,21,22]. Detection using a SiO_2_ OWRRs as sensing elements has also been applied in many fields, such as acoustic wave detection, single-nanoparticle detection and angular rate detection [5,23,24,25,26]. In the early stage, we developed an approach to the design and fabrication of a resonator, with a Q factor of up to 10^6^–10^7^, which was applied to a resonant micro-optic gyroscope and an optoelectronic oscillator [27,28]. Subsequently, optical waveguide membrane-free acoustic sensors based on grooves etched in ring resonators are proposed [29], and the resonators have Q-factors of up to 10^6^. The acoustic wave can be sensed by calculating the shift of the resonant frequency, due to the change in the air refractive index of the groove caused by the acoustic wave.

In previous work, the grooves were only etched in the coupling region of the resonators. Now, an alternative approach to the design and optimization of a high-Q ring resonator for membrane-free acoustic sensors has been given. We have improved the previous SiO_2_ OWRR and designed a semi-buried OWRR (SOWRR) with air as the upper cladding medium, to achieve higher-sensitivity acoustic detection. In this paper, the design parameters of the SOWRR are discussed and analyzed in detail. The optimal values of the air/SiO_2_:Ge/SiO_2_ waveguide resonator length and coupling spacing are obtained as 50 mm and 5.6 μm, respectively, and the Q factor of the SOWRR is as high as 8.33 × 10^6^. Moreover, the membrane-free acoustic sensor based on the SOWRR has a frequency response range of 1 Hz to 1.58 MHz, and a minimum detectable sound pressure of 7.48 Pa. Therefore, it is expected to become one of the most promising candidates for the next-generation acoustic sensor.

## 2. Principles Analysis

The optical membrane-free acoustic sensor employs a SOWRR as sensing element. Different from the traditional OWRR with SiO_2_ as the upper and lower cladding, the SOWRR utilizes air to replace SiO_2_ as the upper cladding medium. The result is that evanescent field can be easily excited when the laser frequency is locked to the resonant frequency point of the SOWRR. The excited evanescent field in the air cladding medium would be modulated by acoustic wave. Subsequently, the acoustic information can be obtained. Here, we take high Q factor and resonance depth as design criteria to give the optimal design parameters of the SOWRR.

**Figure 1:**
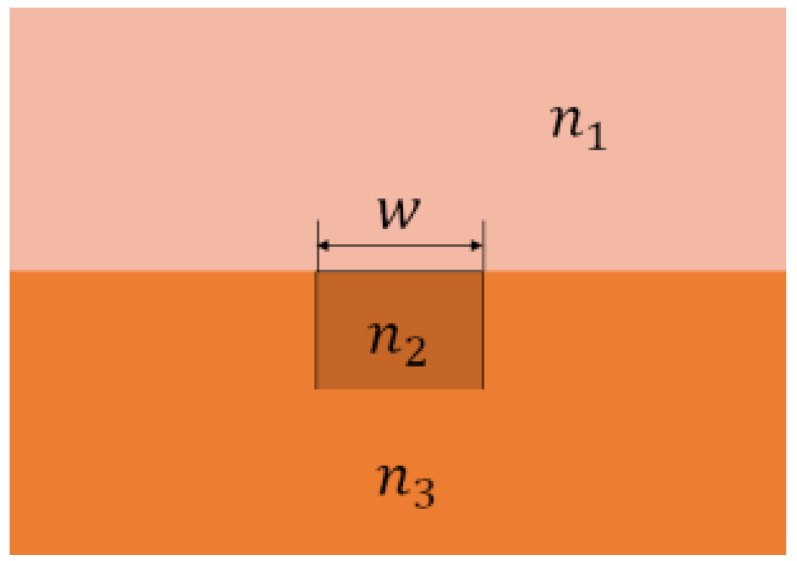
Schematic of a semi-buried optical waveguide illustrating dimensions and material properties. w is the width of optical waveguide; $n_{1}$, $n_{2}$, and $n_{3}$ are the refractive indices of the upper cladding, core layer, and lower cladding, respectively.

Along with the acoustic wave, the density of the medium fluctuates as it compresses or expands. In the absence of dielectric loss, the relationship between acoustic pressure and the variation in density, Δρ, in the dielectric space is established to be

(1) $$p=c^{2}\times \Delta \rho$$

where c denotes the speed of sound in the medium. The propagation properties of light in a medium are related to the refractive index. When the medium is air, the relationship between its density, ρ, and its refractive index, n, can be expressed via the Lorenz–Lorentz equation [30,31]

(2) $$(n^{2}-1)/(n^{2}+2)=\rho \times \alpha$$

where α is a constant denoting the average polarization for isotropic molecules. As for our structure, sound vibration causes a change in the refractive index of the upper cladding (air) as shown in Figure 1. According to Equation (2), the change in refractive index for the air as a result of density is

(3) $$\Delta n={\left(n_{1}^{2}+2\right)}^{2}/6n_{1}\times \alpha \times \Delta \rho$$

Here, c is 340 m/s, α is $1.63\times 10^{-4}$ and the refractive index of the air, *n*_1_, is approximately 1.0003. As can be calculated from Equations (1) and (3), the acoustic pressure action of 1 Pa will produce a 2.12 × 10^−9^ refractive index change.

The structure of the semi-buried optical waveguide (Figure 1) comprises an optical waveguide of width, w, made of a core material of the refractive index $n_{2}$, and an upper and lower cladding layers of refractive indices $n_{1}$ and $n_{3}$. The materials used for the optical waveguide are air/SiO_2_: Ge/SiO_2_. Adopting a first-order model of the TE mode for confined optical wave propagation, we used the effective refractive index method to derive the eigen equation [32,33]

(4) $$\gamma_{2}w=\arctan\left(c_{1}\times \gamma_{1}/\gamma_{2}\right)+\arctan\left(c_{2}\times \gamma_{3}/\gamma_{2}\right)$$

where $c_{1}$ and $c_{2}$ denote the velocities of sound in the upper and lower cladding layers, and

(5) $$\left\{\begin{array}{l} \gamma_{1}=k_{0}\sqrt{n_{eff}^{2}-n_{1}^{2}}\\ \gamma_{2}=k_{0}\sqrt{n_{2}^{2}-n_{eff}^{2}}\\ \gamma_{3}=k_{0}\sqrt{n_{eff}^{2}-n_{3}^{2}} \end{array}\right.$$

(6) $$k_{0}=2\pi /\lambda_{0}$$

in which $n_{eff}$ denotes the effective refractive index of the optical waveguide. $k_{0}$ is the wave number in vacuum. This effective refractive index $n_{eff}$ is determined via Equation (4). As this equation is an implicit equation for $n_{eff}$, there is no algebraic solution. To analyze the acoustic waves, both $n_{2}$ and $n_{3}$ are set as constants, whereas $n_{1}$ and $n_{eff}$ are set as variables. The dependence of $n_{eff}$ on $n_{1}$ from this analysis satisfies a parabolic equation:

(7) $$n_{eff}=k_{1}\times n_{1}^{2}+k_{2}\times n_{1}+k_{3}$$

in which $k_{i}$(i = 1, 2, 3) is a constant, the values of which are obtained via curve fitting. A variation in the refractive index of the upper cladding, Δn, produces a variation in the effective refractive index, $\Delta n_{eff}$, which is described by

(8) $$\Delta n_{eff}=(2\times k_{1}\times n_{1}+k_{1}\times \Delta n+k_{2})\cdot \Delta n$$

**Figure 2:**
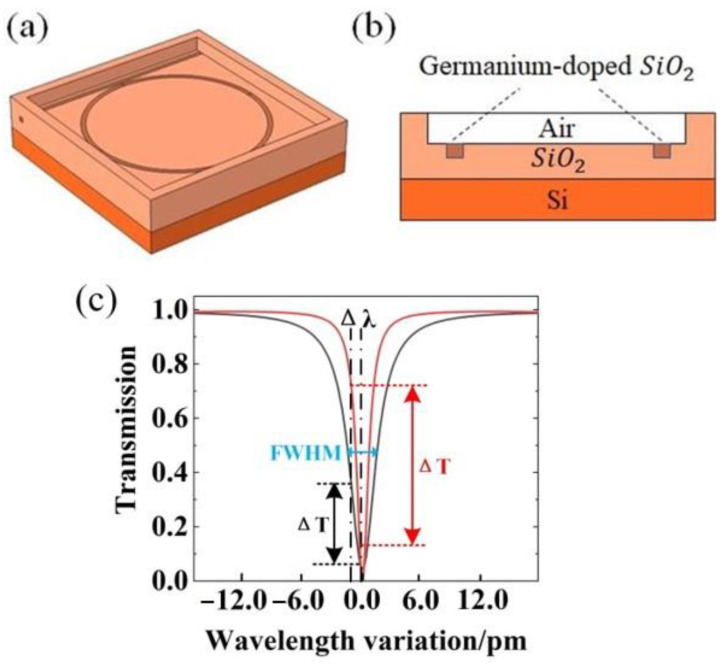
(**a**) Structure diagram and (**b**) cross-section of a semi-buried optical waveguide resonator. (**c**) Resonance curves for two SOWRRs with different FWHM values.

The basic configuration of the SOWRR is a three-dimensional structure as shown in Figure 2a, in which the SiO_2_ OWRR structure (Figure 2b) is etched to form a cladding of air. Acoustic waves propagate in a direction perpendicular to the ring resonator. This structure can stimulate the evanescent field of the OWRR, in which evanescent field light waves can drift in resonant frequency due to changes in air density caused by the acoustic wave. This modification results in an acoustic sensor with high sensitivity. The light transmitted in the SOWRR must satisfy the resonance condition, and the wavelength, λ, at resonance is given by

(9) $$L\times n_{eff}=m\times \lambda$$

where L denotes the length of the cavity, and m is the resonance mode. The shift in the resonance wavelength, Δλ, is obtained from

(10) $$L\times \Delta n_{eff}=m\times \Delta \lambda$$

Applying the transfer matrix method [27], the transfer function of the SOWRR is expressed as

(11) $$T=\frac{t^{2}+a^{2}-2ta\cos(2\pi n_{eff}L/\lambda )}{1+t^{2}a^{2}-2ta\cos(2\pi n_{eff}L/\lambda )}$$

where t denotes the coefficient of transmission, and a is the transmission factor for light propagating in the ring resonator for one round. The SOWRR resonance curves for different full-width at half-maximum (FWHM) settings (Figure 2c) illustrate the point that ΔT is different despite Δλ being the same and the FWHM and the working wavelength being different. In general, the wavelength corresponding to the maximum slope of the resonance curve is chosen as the center of the wavelength shift to obtain the maximum ΔT on the resonance curve. With this simulation, obtaining the maximum ΔT requires finding the smallest FWHM. The FWHM subsequently determines the Q factor of the SOWRR via

(12) $$Q=f_{0}/FWHM$$

with $f_{0}$ denoting the central frequency of the laser. FWHM in Equation (12) is given by

(13) $$FWHM=c/(n_{eff}\pi L)\arccos2ta/(1+t^{2}a^{2})$$

where c denotes the speed of light in a vacuum.

A decrease in FWHM is associated with an increase in the Q factor, as well as an increase in ΔT. For the SOWRR acoustic sensor, a high sensitivity corresponds to a larger ΔT, which demands a high Q factor. Therefore, finding the appropriate design of the SOWRR is necessary to attain a maximum Q factor.

## 3. Results and Discussion

From the above analysis, we conclude that the cavity length and coefficient of transmission are key parameters to achieve the objectives for our SOWRR. They determine the Q factor that must be optimized to realize a high-performing acoustic sensor.

**Figure 3:**
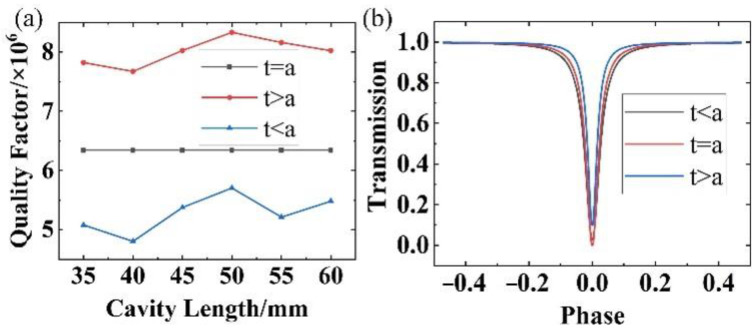
(**a**) Q factor of the SOWRR for different cavity lengths corresponding to t=a, t > a, and t < a. (**b**) Resonance curve of a 50 mm long SOWRR corresponding to t < a, t=a, and t > a.

The relationship between the cavity length of the SOWRR and the Q factor in the under-coupled, t>a, critically coupled, t=a, and over-coupled, t<a, regimes for various parameter settings (Table 1) are displayed in Figure 3a. They indicate that the Q factor varies little in value with an increasing cavity length, particularly in the critically coupled regime. From this aspect, the resonance linewidth of a SOWRR in the under-coupled regime is obviously narrower than that in the other two regimes. However, the larger the cavity length, the larger the resonator volume. For the same value of the coefficient of transmission, a cavity of length 50 mm achieves the highest Q factor due to the above trade-off effects.

With a cavity length of 50 mm for the SOWRR, resonance curves were calculated for the three regimes (Figure 3b). The under-coupled regime yields the smallest resonance linewidth, but the corresponding resonance depth is reduced. The expression for the resonance depth is

(14) $$h=1-{\left(\left(t-a)/(1-ta\right)\right)}^{2}$$

The critical regime (t=a) provides the largest resonance depth; the other two regimes reduce the resonance depth. Nonetheless, in practical applications, the resonance depth can be sacrificed if the aim is to attain a high Q factor.

**Figure 4:**
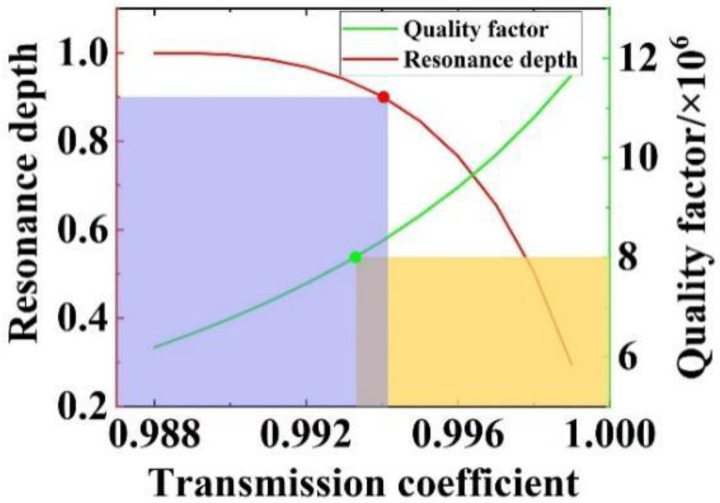
The resonance depth, *h*, and Q factor versus the coefficient of transmission, t (L = 50 mm, a = 0.9886).

For an under-coupled resonator with a cavity length of 50 mm, resonance depths obtained from Equation (14) for different values of the coefficient of transmission, t, are calculated (Figure 4). The corresponding Q factors are also presented for comparison.

With the aim of attaining a resonance depth of above 0.9 and a Q factor of no less than 8 × 10^6^ for our SOWRR, we have highlighted the regions in purple and yellow, respectively. Their region of intersection satisfies both criteria simultaneously. A coefficient of transmission of a value of 0.994 is optimal for the SOWRR.

**Figure 5:**
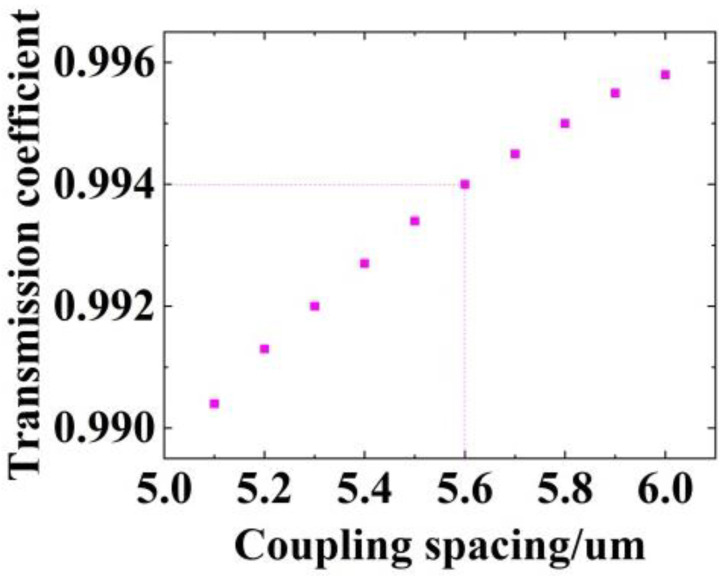
Coefficient of transmission for the ring resonator for different coupling spacings.

**Figure 6:**
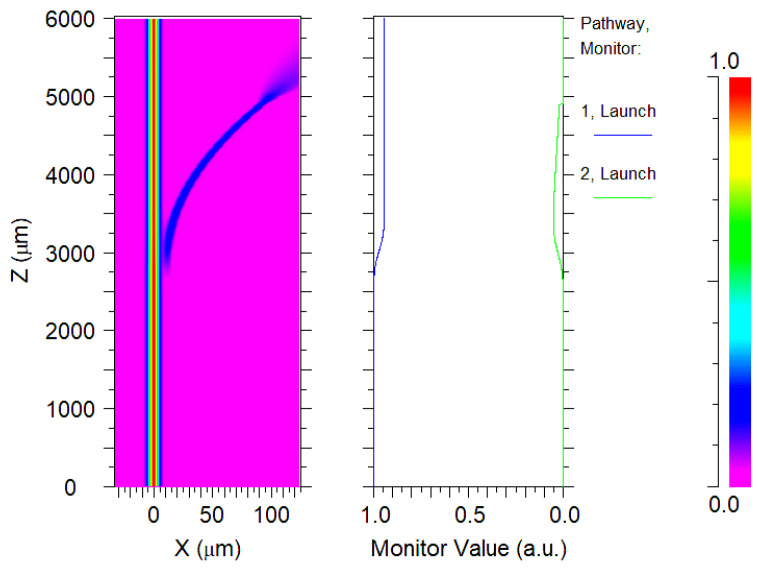
Electric field distribution and transmittance curve for a cavity with a 5.6 µm coupling spacing.

The dependence of the coefficient of transmission on the coupling spacing (Figure 5) shows that the coefficient of transmission with a value of 0.994 corresponds to a coupling spacing of 5.6 µm. Configuring a resonator with this spacing, we generated its electric field distribution (Figure 6) along with the transmitting curves of a straight waveguide (labeled Launch 1) and the ring waveguide (labeled Launch 2).

**Figure 7:**
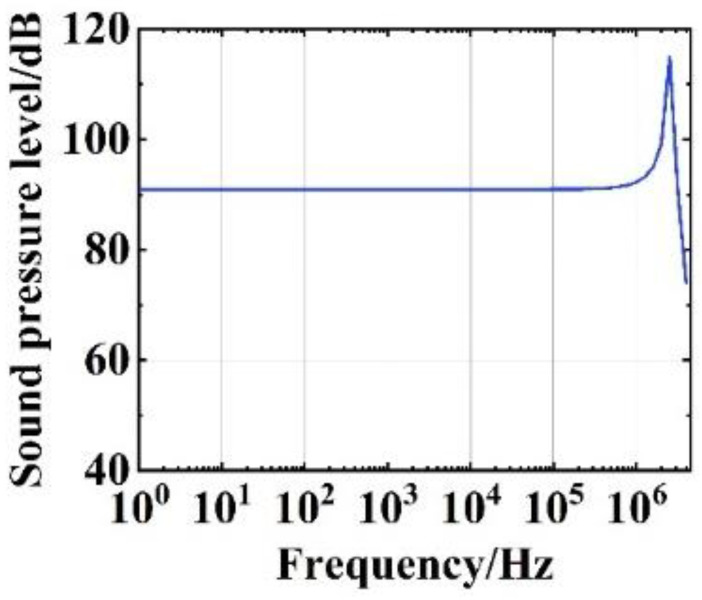
Frequency response curve of the semi-buried ring resonator acoustic sensor.

**Figure 8:**
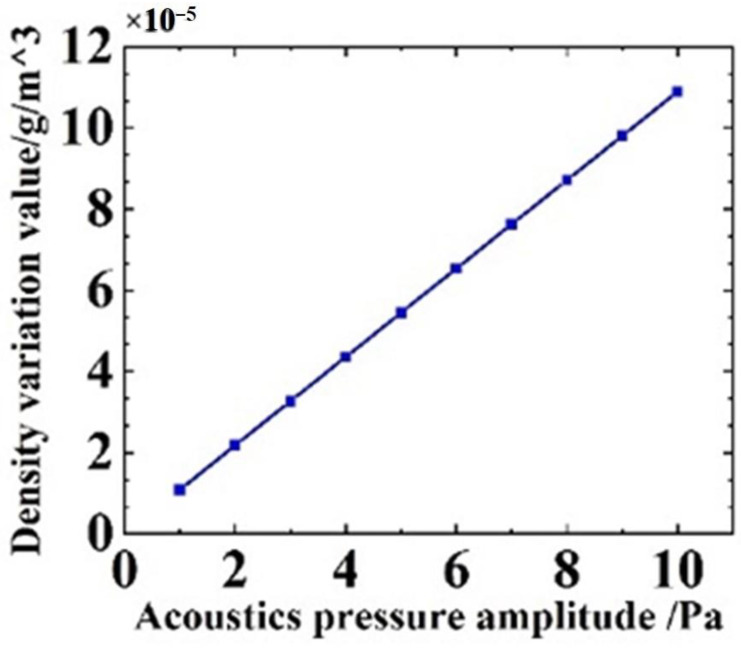
Variation in the density against the amplitude of the acoustic pressure (*f* = 1.2 MHz).

To study the performance of the SOWRR acoustic sensor when subjected to sound pressure, frequency response analysis and air density analysis were performed in a finite element simulation (see Figure 7 and Figure 8). For the settings given in Table 1, based on the lowest natural frequency of the structure, 2.45 MHz, the frequency response curve of the SOWRR acoustic sensor (Figure 7) exhibits a flat-frequency response in the range from 1 Hz to 1.58 MHz. The peak of the curve corresponds to the lowest natural frequency of the SOWRR. The variation in the acoustic pressure amplitude with the density of the upper cladding medium at a frequency of 1.2 MHz (Figure 8) shows a positive correlation.

The sensitivity of the designed acoustic sensor is mainly related to two factors: the change in the refractive index caused by the sound pressure, and the frequency shift of the transmission spectrum caused by the change in the refractive index, in which the frequency shift of the transmission spectrum caused by the change in the refractive index is a directly influencing factor.

When the external sound wave acts on the air medium, the air density is changed due to the action of the sound pressure. Since the acoustic wave is a periodic sound pressure change, the variation period of the air density is also consistent with the pressure change period of the acoustic wave.

According to the basic theory of acoustics, the sound pressure’s sensitivity can be expressed as the frequency shift, Δf, of the resonance spectrum caused by sound pressure, p, as shown in (15):

(15) $$S=\frac{\Delta f}{p}$$

By calculating the parameters of the ring waveguide cavity designed in this paper, it can be concluded that the sound pressure sensitivity is 133.75 MHz/Pa.

The minimum detectable acoustic pressure of an acoustic sensor is also an important index to evaluate its performance. If a narrow-linewidth laser is used as a light source for a SOWRR acoustic sensing system, the minimum detectable acoustic pressure is determined via the ratio of the laser linewidth to the frequency shift per 1 Pa of acoustic pressure. From a calculation, the minimum detectable acoustic pressure for the SOWRR was 7.48 µPa when using a laser beam with a linewidth of 1 kHz. The sensitivity of the structure depends on the refractive index and width of the waveguide, the length of the cavity of the ring resonator, and the coupling spacing. Acoustic sensors with different sensitivities can be obtained by designing SOWRRs of different sizes. Therefore, the proposed acoustic sensor based on the SOWRR has wide application flexibility.

## 4. Conclusions

In summary, we developed a SOWRR structure designed for acoustic sensors to provide a wide-frequency response and high-sensitivity detection. The structure utilizes the upper cladding of the waveguide as a sensing element of acoustic pressure. The density of the coated gas medium on the waveguide cavity is changed, and the effective refractive index of the waveguide is changed, resulting in a shift in resonator frequency. The optimal values for the cavity length and coupling spacing of the air/SiO_2_:Ge/SiO_2_ waveguide resonator were found to be 50 mm and 5.6 µm. With a laser linewidth of 1 kHz, the minimum detectable sound pressure was 7.48 µPa. The frequency response of the SOWRR is in the range 1 Hz to 1.58 MHz. By selecting suitable coupling spacing and the cavity length of a SOWRR, acoustic sensors appropriate for different acoustic detection fields can be obtained.

## Figures and Tables

**Table 1:** Parameters for the SOWRR.

Characteristics	Symbols	Values
Wavelength of light	λ	1.55 µm
Frequency linewidth of laser	Δf	1 kHz
Effective refractive index	$n_{eff}$	1.4486
Transmission loss per unit Length	α	0.02 dB/cm
Cavity length	L	0.05 m
Transmission factor	a	0.9886

## Data Availability

The data presented in this study are available upon request from the corresponding author.
